# Supplementary material for: Functional border-associated macrophages limit Alzheimer’s Disease progression
Source: bioRxiv. 2026 Feb 3:2026.01.31.703045. Preprint. [Version 1] doi: 10.64898/2026.01.31.703045 (PMC12889433; doi:10.64898/2026.01.31.703045)
Supplement: Supplement 1 [file nihpp2026.01.31.703045v1-supplement-1.pdf]

bioRxiv preprint doi: https://doi.org/10.64898/2026.01.31.703045; this version posted February 3, 2026. The copyright holder for this preprint (which was not certified by peer review) is the author/funder, who has granted bioRxiv a license to display the preprint in perpetuity. It is made available under aCC-BY 4.0 International license.

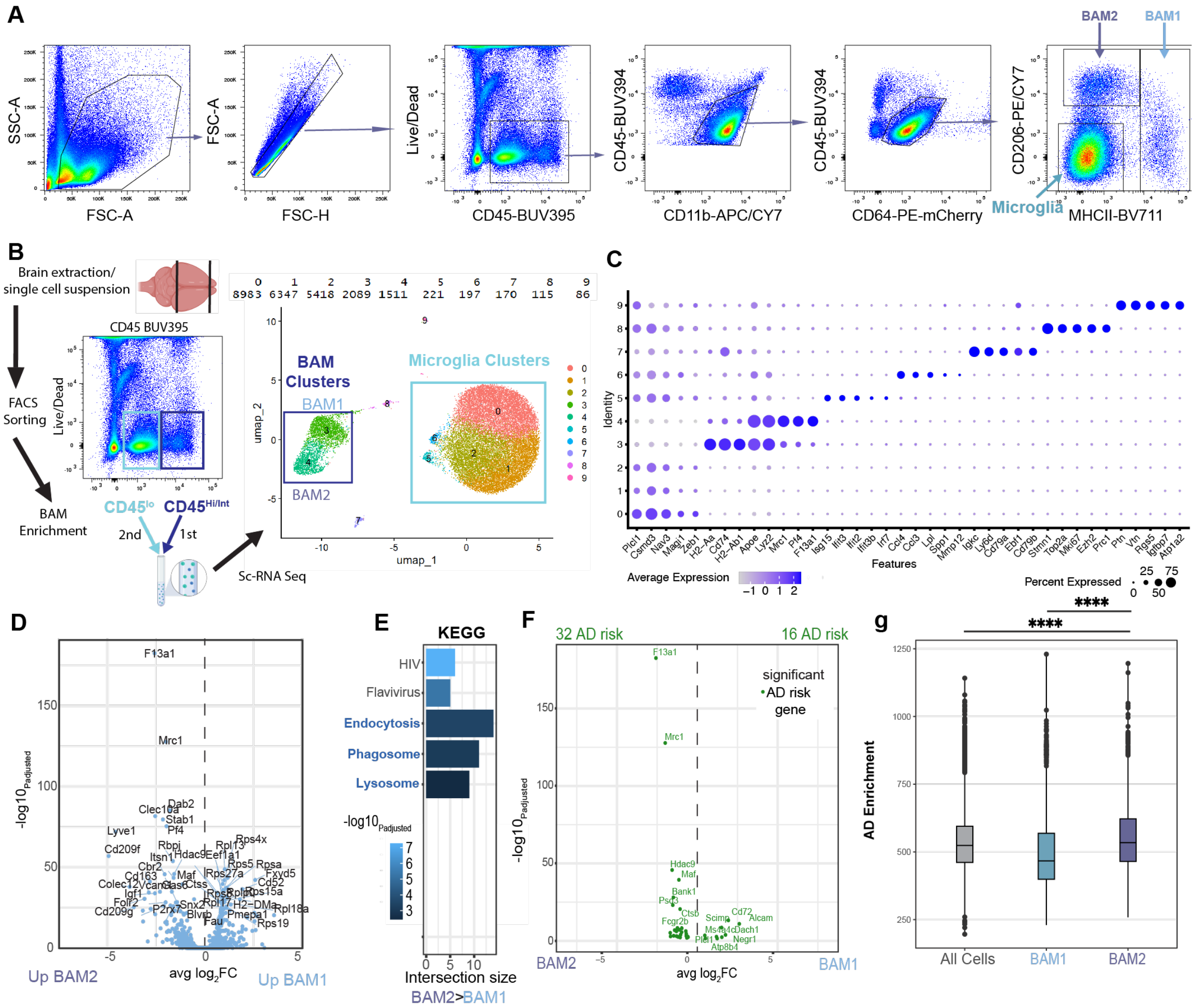

Figure S1. CD206^{Hi} BAMs (BAM2) are highly phagocytic and enriched in AD risk. Related to Figure 1. A, Gating strategy for MG and BAM subtypes. Brain resident macrophages were gated as CD45^{+}CD11b^{+}CD64^{+}. Within this gate Microglia were gated as CD206^{-}MHCII^{-}, BAM2 were gated as CD206^{+}MHCII^{-}, and BAM1 were gated as MHCII+. **B**, Pipeline and BAM enrichment strategy for brain immune cell single cell RNA-Seq with corresponding UMAP defining cellular clusters and (above) number of cells within each cluster. **C**, Marker genes defining each cluster from (**B**). **D**, Volcano plot with all significant genes ($P_{adjusted}$<.05) enriched in BAM2 vs BAM1. **E**, KEGG analysis of all pathways enriched in BAM2>BAM1. **F**, Whisker plot of all genes containing associated AD GWAS alleles (MONDO_0004975) enriched in BAM2 vs BAM1 clusters with (above) corresponding counts within each cell group. **G**, Per-cell AD enrichment score within BAM2, BAM1, and all cell aggregate clusters. Wilcoxon Rank Sum Test, P****<.0001. Inset box plots with median and 25th and 75th percentiles. UMAP= uniform manifold approximation and projection.

bioRxiv preprint doi: https://doi.org/10.64898/2026.01.31.703045; this version posted February 3, 2026. The copyright holder for this preprint (which was not certified by peer review) is the author/funder, who has granted bioRxiv a license to display the preprint in perpetuity. It is made available under aCC-BY 4.0 International license.

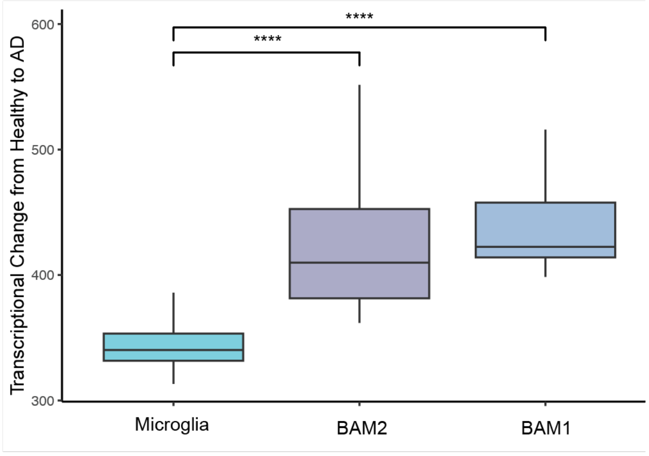

Figure S2. AD changes BAMs transcriptionally. Related to Figure 2. Quantification of the Euclidean distance of transcriptional change within brain macrophage subtypes between healthy and AD conditions with corresponding Chi-squared p-value. ****P<.0001, Pairwise t-test with Benjamini-Hochberg adjustment. Box plots= median + 25/75th percentile.

bioRxiv preprint doi: https://doi.org/10.64898/2026.01.31.703045; this version posted February 3, 2026. The copyright holder for this preprint (which was not certified by peer review) is the author/funder, who has granted bioRxiv a license to display the preprint in perpetuity. It is made available under aCC-BY 4.0 International license.

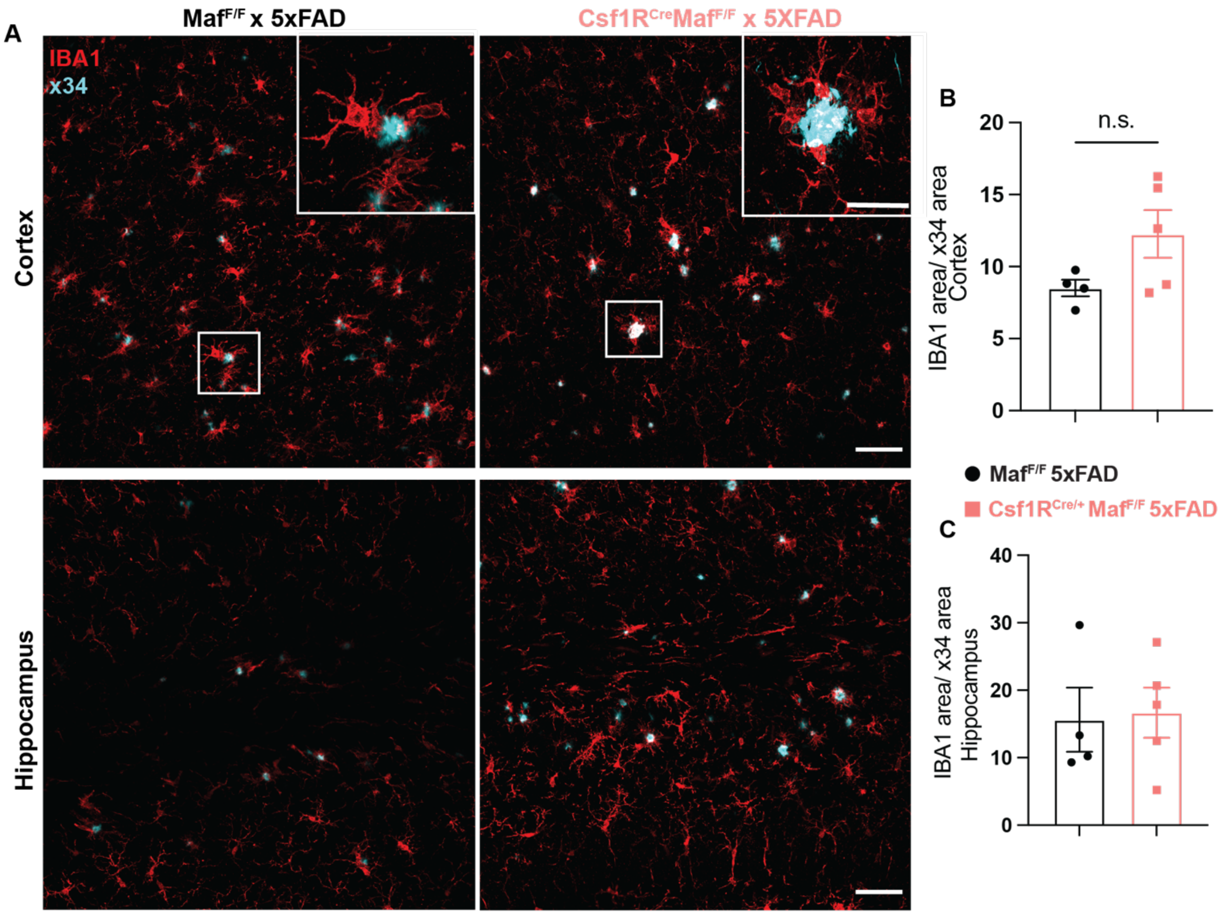

Figure S3. Microglia from Csf1R$^{Cre/+}$*Maf*$^{F/F}$ mice are reactive to amyloid. Related to Figure 3. A, IBA1^{+} (red) microglia staining and association with X-34 staining (cyan) of compacted amyloid in sections of BAM2-depleted Csf1R$^{Cre/+}$*Maf*$^{F/F}$ 5xFAD mice and *Maf*$^{F/F}$ 5xFAD controls from similar regions of cortex and hippocampus. **B-C**, quantification IBA1 area normalized to amyloid area in (**B**) cortex and (**C**) hippocampus (n=4 *Maf*$^{F/F}$ 5xFAD, 5 Csf1R$^{Cre/+}$*Maf*$^{F/F}$ 5xFAD mice). Two-tailed unpaired student's t-test. Scale bar=100µm low magnification images, 50µm for high magnification insets.

bioRxiv preprint doi: https://doi.org/10.64898/2026.01.31.703045; this version posted February 3, 2026. The copyright holder for this preprint (which was not certified by peer review) is the author/funder, who has granted bioRxiv a license to display the preprint in perpetuity. It is made available under aCC-BY 4.0 International license.

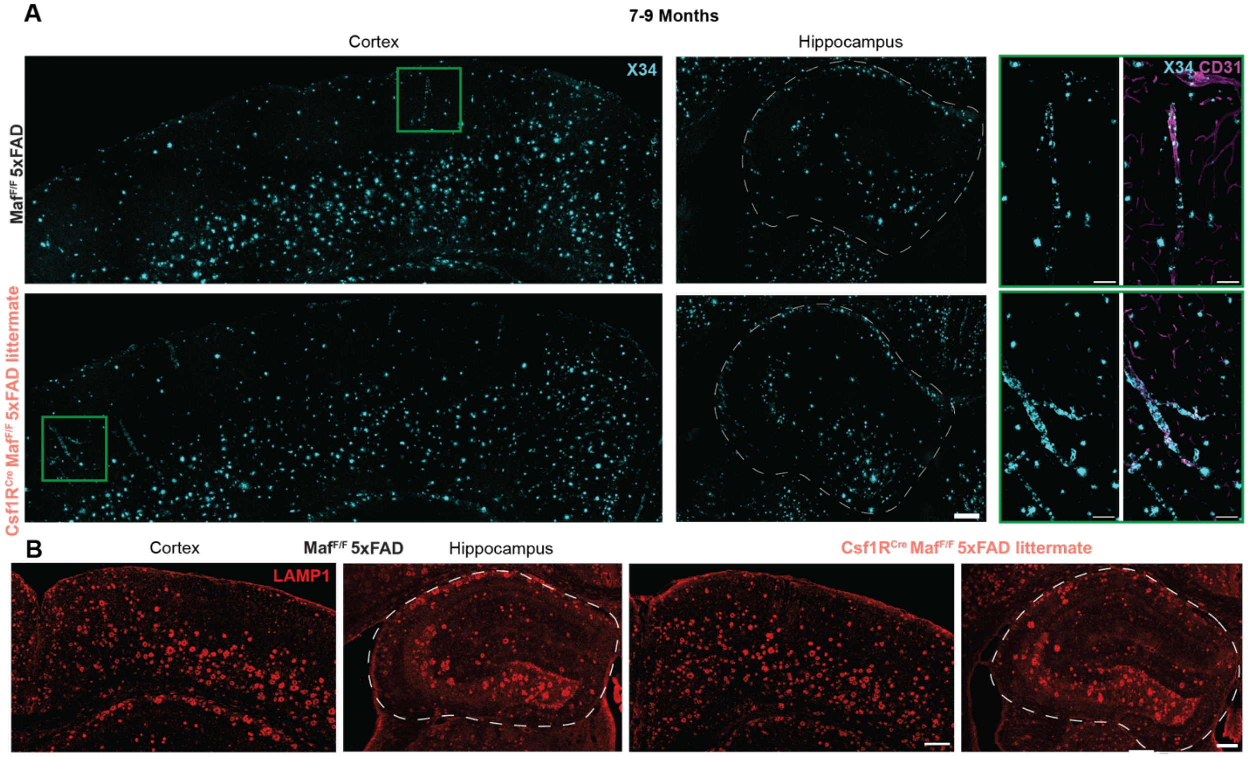

Figure S4. BAM2 depletion has minimal effect on Aβ load and neurodystrophy at late disease stages. Related to Figure 4. A. Representative micrographs of X-34 amyloid staining in cortex and hippocampus in 7-9 month old Cre$^+$ (BAM2 depleted) and Cre$^-$ 5xFAD littermates with regions of interest (ROIs, green boxes) depicting CAA in pial and penetrating CD31$^+$ blood vessels in cortex. **B.** As with **A** but for LAMP1 neurodystrophy marker. Scale bars=200µm large images, 50µm high mag ROIs.

bioRxiv preprint doi: https://doi.org/10.64898/2026.01.31.703045; this version posted February 3, 2026. The copyright holder for this preprint (which was not certified by peer review) is the author/funder, who has granted bioRxiv a license to display the preprint in perpetuity. It is made available under aCC-BY 4.0 International license.

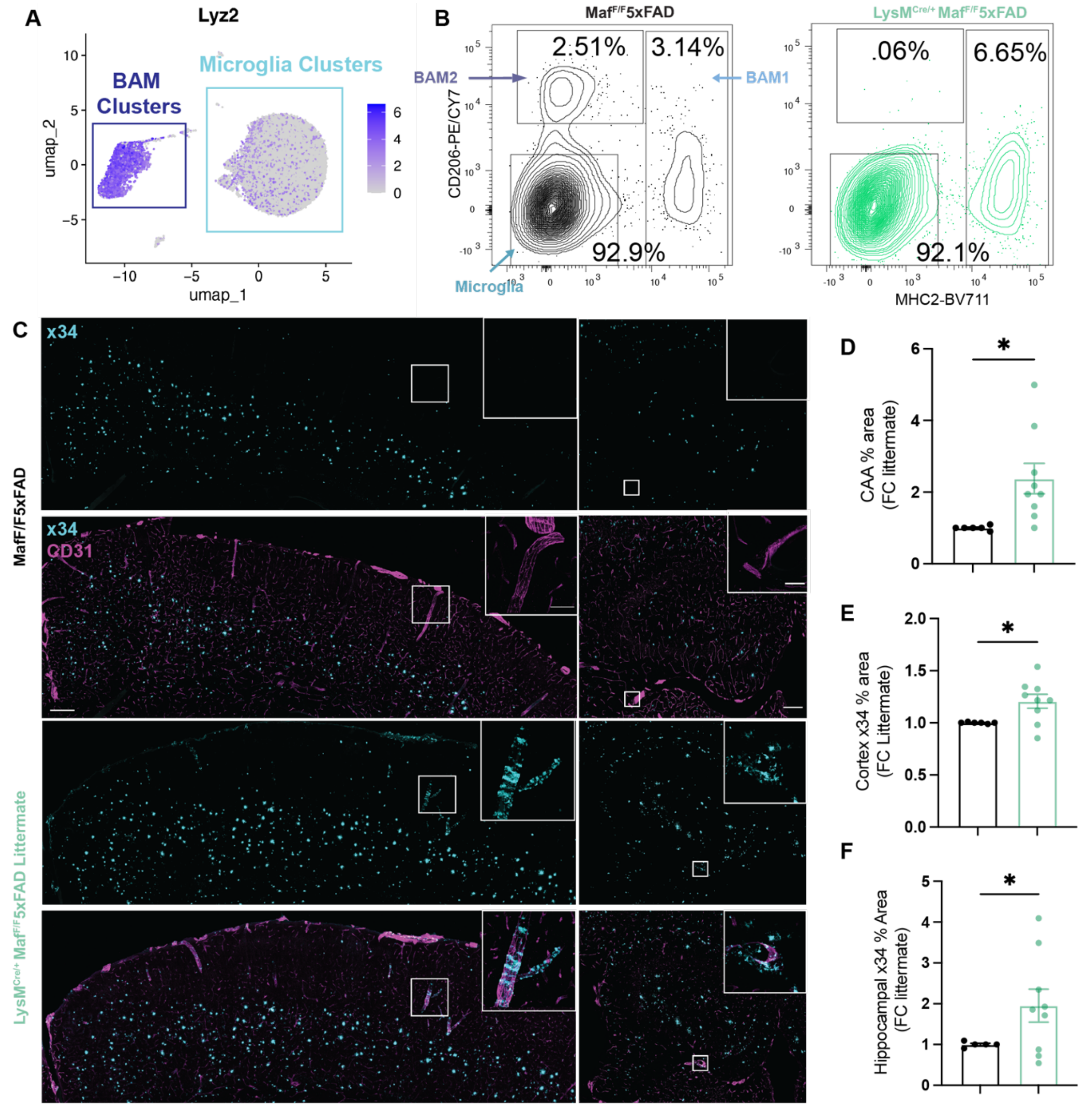

Figure S5. LysM$^{Cre/+}$*Maf*$^{F/F}$5xFAD have early acceleration of AD phenotypes. Related to Figures 3 and 4. A, Heatmap of relative *Lys2* (*LysM*) expression per cell in clusters detailed in **Fig. S1b**. **B**, Representative contour plots of brain macrophage populations (BAM2, top box; BAM1, right box; MG, bottom left box) with quantification of percentage of CD64^{+} gated cells. Note an increase in percentage of BAM1, which does not reflect a conversion of BAM2 into BAM1, but a rebound of BAM2, which are repopulated from monocytes [32]. **C**, Representative micrographs depicting X-34 amyloid staining and CD31^{+} endothelial cells in LysM$^{Cre/+}$*Maf*$^{F/F}$ 5xFAD and *Maf*$^{F/F}$ 5xFAD mice with high magnification insets depicting prominent CAA in LysM$^{Cre/+}$*Maf*$^{F/F}$ 5xFAD mice. **D-F**, Quantification of (**D**) CAA % blood vessel area, (**E**) cortical, and (**F**) hippocampal X-34 % area normalized to same sex littermate controls. Mean+/- S.E.M. Scale bars=200μm large images, 50μm insets.

bioRxiv preprint doi: https://doi.org/10.64898/2026.01.31.703045; this version posted February 3, 2026. The copyright holder for this preprint (which was not certified by peer review) is the author/funder, who has granted bioRxiv a license to display the preprint in perpetuity. It is made available under aCC-BY 4.0 International license.

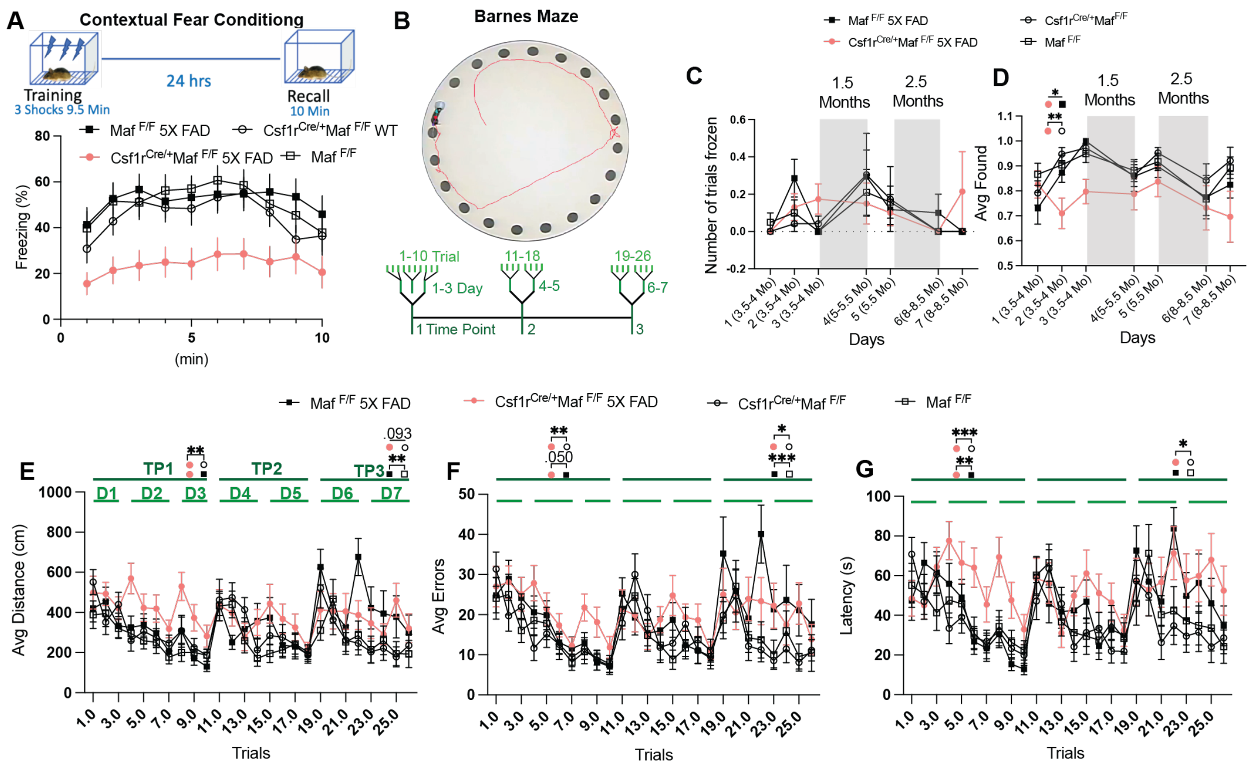

Figure S6. Behavioral analysis in BAM2 depleted AD mice. Related to Figure 4. A, Contextual Fear Conditioning experiment recall (**from Fig. 4N**) binned by 1-minute intervals. Significant 5xFAD x Minute interaction (Huynh-Feldt: $F(6.982,432.880) = 2.397$, $p=0.021$) and main effect of Cre ($F(1,62) = 7.101$, $p=0.01$). See main figure legend for n values. **B-G**, (**B**) Barnes Maze experiment (**from Fig. 4P)** with (**C**) average number of trials frozen per animal by day, (**D**) avg trials with hole found by day (Cre x 5xFAD x Day interaction ($F(6,1858) = 3.766$, $p < 0.001$) (**E**) avg Distance by trail, (**F**) avg Errors by trial, and (**G**) avg Latency per animal by trial. Two-way repeated measures ANOVA with Sidak's multiple comparisons test. See *Materials and Methods* for n values. Significant pairwise comparisons and select p values within time points indicated by shapes associated with respective genotypes in the panel legend. Linear Mixed Effects Model revealed a main effect of Trial across Barnes Maze performance measures: (**D**) $F(3,1856) = 17.854$, $p<0.001$), (**E**) $F(3,1856) = 13.585$, $p<0.001$), (**F**) $F(3,1856) = 9.188$, $p<0.001$). (**C**) Sidak comparisons also revealed that Cre^{+}5xFAD mice had a reduced ability to find the escape hole across days (all $p<0.05$), unlike all other groups. *P<.05,**P<.01, ***P<.001. TP=Time Point (months), D=days.

bioRxiv preprint doi: https://doi.org/10.64898/2026.01.31.703045; this version posted February 3, 2026. The copyright holder for this preprint (which was not certified by peer review) is the author/funder, who has granted bioRxiv a license to display the preprint in perpetuity. It is made available under aCC-BY 4.0 International license.

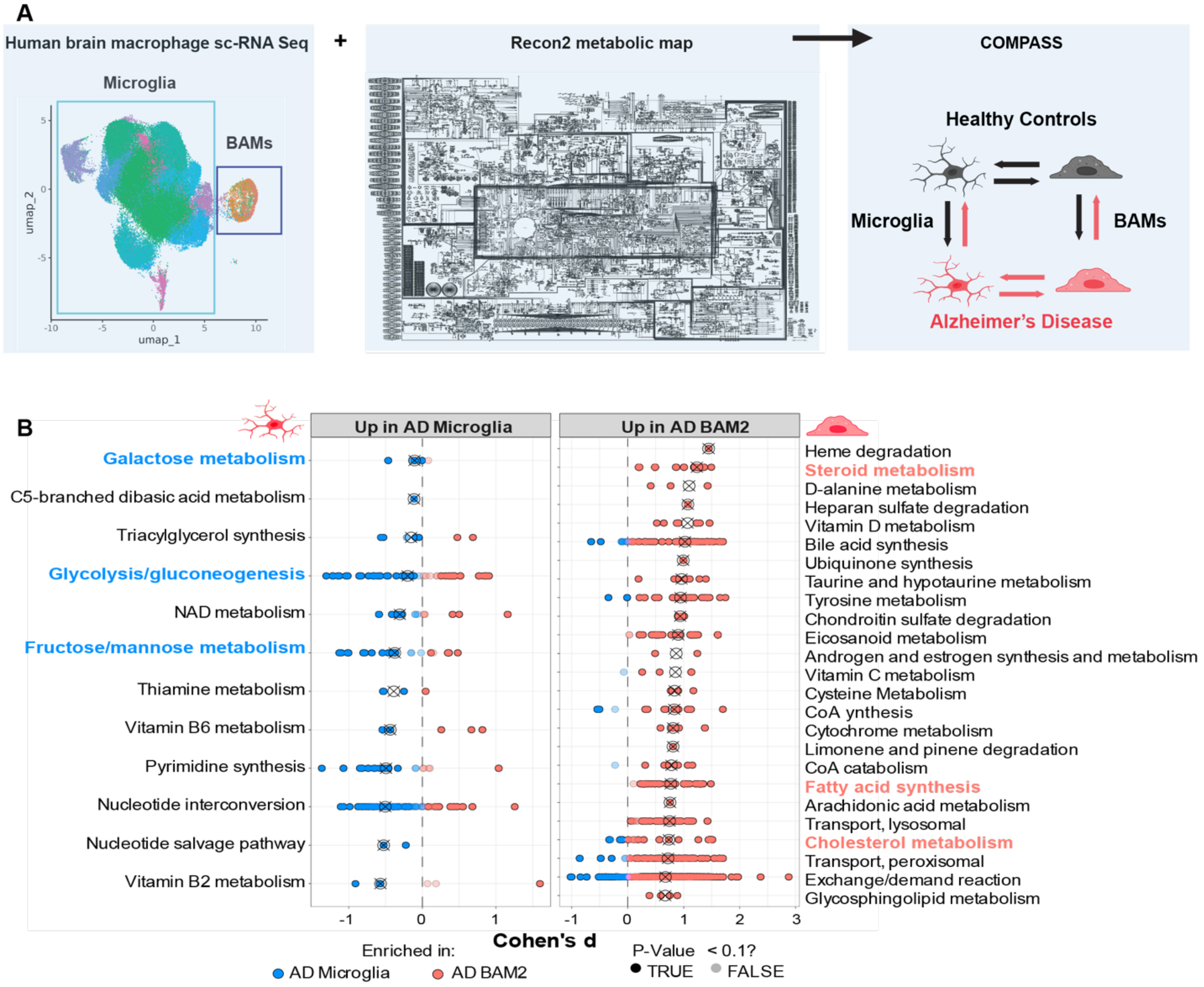

Figure S7. Human BAMs differ metabolically from microglia in health and AD. Related to Figure 6. A, Schematic of pipeline for *in silico* COMPASS analysis[54] on aggregate human brain macrophages from control and AD patients. **B**, All discovered subsystems (Cohen's d>.1) enriched in human microglia vs BAM2s from AD patients Colored subsystem names represent pathways discussed in main text. Circles with x=median enrichment for the respective subsystem. Individual circles=individual reactions within the subsystem that when shaded dark =$P_{adjusted}$<.1 (Benjamini-Hochberg adjusted).

bioRxiv preprint doi: https://doi.org/10.64898/2026.01.31.703045; this version posted February 3, 2026. The copyright holder for this preprint (which was not certified by peer review) is the author/funder, who has granted bioRxiv a license to display the preprint in perpetuity. It is made available under aCC-BY 4.0 International license.

Table S1. (separate file)

Mouse Single-Cell RNA-seq comparisons.

Table S2. (separate file)

Human brain macrophage cluster markers.

Table S3. (separate file)

Compass individual reaction comparisons.
